# Drug Resistance and Virological Failure among HIV-Infected Patients after a Decade of Antiretroviral Treatment Expansion in Eight Provinces of China

**DOI:** 10.1371/journal.pone.0166661

**Published:** 2016-12-20

**Authors:** Zhongbao Zuo, Shu Liang, Xianguang Sun, Scottie Bussell, Jing Yan, Wei Kan, Xuebing Leng, Lingjie Liao, Yuhua Ruan, Yiming Shao, Hui Xing

**Affiliations:** 1 State Key Laboratory of Infectious Disease Prevention and Control, National Center for AIDS/STD Control and Prevention, Chinese Center for Disease Control and Prevention, Collaborative Innovation Center for Diagnosis and Treatment of Infectious Diseases, Beijing, China; 2 Sichuan Center for Disease Control and Prevention, Chengdu, China; 3 Guizhou Center for Disease Control and Prevention, Guiyang, China; 4 Vanderbilt Institute for Global Health, Nashville, Tennessee, United States of America; National and Kapodistrian University of Athens, GREECE

## Abstract

**Background:**

China’s National Free Antiretroviral Treatment Program (NFATP) has substantially increased the survival rate since 2002. However, the emergence of HIV drug resistance (HIVDR) limits the durability and effectiveness of antiretroviral treatment (ART) in at risk patients.

**Method:**

A cross-sectional survey was conducted among patients having received a median of 13.9 months of ART in eight provinces in China. Demographic and clinical information was collected, and venous blood was sampled for CD4 cell counts, measurement of the HIV viral load (VL), and HIV drug resistance (HIVDR) genotyping. Possible risk factors for HIVDR were analyzed by the logistic regression model.

**Results:**

The study included 765 patients. Among them, 65 patients (8.5%) had virological failure (VLF) defined as ≥1,000 copies/ml. Among the individuals with VLF, 64 were successful genotyped, and of these, 33 had one or more HIVDR mutations. The prevalence of HIVDR mutations among patients receiving first-line ART was 4.3% (33/765). All of the patients with HIVDR mutations were resistant to non-nucleoside transcriptase inhibitors, 81.8% were resistant to nucleoside reverse transcriptase inhibitors, and only 3% had mutations that caused resistance to protease inhibitors. Having lower ratios of drug intake in the past month and dwelling in two southwestern provinces were factors independently associated with the emergence of HIVDR.

**Conclusion:**

Most patients receiving first-line ART treatment achieved sound virological and immunological outcomes. However, poor adherence is still a key problem, which has led to the high rate of HIVDR. It was notable that the proportion of drug resistance widely varied among the provinces. More studies are needed to focus on adherence.

## Introduction

Since its development in 1996, combination antiretroviral therapy (cART) has significantly improved the quality of life of HIV-infected persons and dramatically lowered their morbidity and mortality [1–5]. It has become widely available in most resource-limited or developing countries since the WHO launched the “3 by 5” initiative in 2003. It was reported at the world AIDS day 2015 that 16 million people were receiving antiretroviral treatment and 7.8 million HIV-related deaths had been averted between 2000 and 2015.

In China, the National Free Antiretroviral Treatment Program (NFATP) was begun in 2002, after a pilot study [6]. As a public health approached ART program, NFATP has proved to have efficiently reduced mortality among HIV-infected Chinese patients [7–12]. The development of NFATP was in three phases. The first phase was from 2002 to 2005, the second phase,also called as the first scale-up phase, was between 2005 and 2007, and the third phase which was the further scale-up and standardization phase was from 2008 onwards [13]. Significant policy changes in the third phase included scale-up HIV testing among key populations and immediate initiation of ART in China. By the end of 2014, more than 363,000 patients in China had received ART [14]. However, concerns for the emergence of drug resistance grew during the rapid ART expansion in China. In this study, we conducted a survey of acquired HIVDR based on the WHO HIVDR surveillance protocol in order to assess the level of virological suppression and drug resistance during these years in China. Our findings would provide valuable implications for good practice of planning treatments for all people living with HIV.

## Methods

### Study design and study population

We conducted a cross-sectional survey in eight provinces or cities of China: Beijing, Jilin, Hunan, Guangxi, Sichuan, Guizhou, Yunnan and Xinjiang Province. The survey protocol was taken from the WHO recommended cross-sectional survey on acquired HIVDR in adult patients receiving ART. Patients included were 18 years or older, had begun free ART treatment in 2013, and had received first-line ART for 9–18 months at enrollment. Eligible patients were enrolled at routine clinic visits in 2014. All participants provided written informed consent.

### Ethics approval

Institutional review board approval was granted by National Center for AIDS/STD Control and Prevention (NCAIDS), Chinese Center for Disease Control and Prevention (China CDC).

### Data collection

An interview-administered questionnaire (S1 File) was used for data collection. The questionnaire was administered face to face by trained local health staff in a private room. Data on demographic characteristics, ART treatment, and medicine adherence were collected during the interview.

### Laboratory tests

Blood specimens were collected after the interview. CD4+ T cells were quantified using flow cytometry at local CDCs within 12 hours. Plasma was isolated and sent under cold chain to the laboratory at NCAIDS, China CDC where the HIV viral load was measured. Viral suppression was defined as an HIV RNA level <1000 copies/ml. In samples with a viral load ≥1000 copies/ml, HIV drug resistance genotyping was performed at the NCAIDS laboratory by using an in-house method [15, 16]. A drug resistance mutation was identified and interpreted by using the algorithm of the Stanford HIV Drug Resistance Database (http://hivdb.stanford.edu/pages/algs/sierra_sequence.html). HIV drug resistance mutations were defined as those conferring low-, intermediate, or high- level resistance [17, 18].

### Statistical analysis

All questionnaire data were double-entered using Epidata 3.1 (The Epidata Association Odense, Denmark). Statistical Analyses (S1 Table) were performed using SAS V9.4 (SAS Institute Inc, Cary, North Carolina, USA). Univariate logistic regression models were constructed to explore factors associated with drug resistance. A stepwise multivariate logistic regression model was constructed to select the variables that were independently associated with drug resistance. A P value <0.05 was considered statistically significant, and all tests were two-sided.

## Results

### Demographic characteristics

This survey included 765 patients having received ART for 9–18 months (Table 1). The mean age was 44.7 years. The majority of the subjects were male (71.4%), of Han ethnicity (79.3%), and had received Junior high school or lower education (70.7%). 42.5% were married and 35.3% were farmers. About two-thirds of the patients were infected by heterosexual transmission, followed by homosexual contact (19.2%), and intravenous drug use (8.5%). Most patients started ART with first-line regimens that included AZT+3TC+EFV/NVP (56.0%), D4T+3TC+EFV/NVP (7.6%), or TDF+3TC+EFV/NVP (29.9%). 8.2% of the patients initiated ART with PI (LPV/r)-based second-line regimens. At the time of the survey, the median duration of ART was 13.9 months (interquartile range [IQR] 12.6–15.0).

**Table 1:** Characteristics of HIV patients receiving ART in China.

Characteristics	Number	Percentage (%)
Total	765	
Age (mean ± SD, years)	44.7±13.4	
Sex		
Male	546	71.4
Female	219	28.6
Ethnicity		
Han	607	79.3
Minorities	158	20.7
Education		
Illiterate	32	4.2
Primary school	189	24.7
Junior high school	320	41.8
High school	121	15.8
Junior college or higher	103	13.5
Married		
Yes	325	42.5
No	440	57.5
Occupation		
Farmer	270	35.3
Other	495	64.7
HIV transmission route		
Heterosexual intercourse	517	67.6
Homosexual intercourse	147	19.2
Drug injection	65	8.5
Other	36	4.7
CD4 count before ART		
0–199	347	45.4
200–349	286	37.4
349 or above	132	17.2
CD4 count at survey		
0–199	277	36.2
200–349	202	26.4
349 or above	286	37.4
Duration of ART (months) median, IQR	13.9, 12.6–15.0	
Missed dose in the past month		
Yes	45	5.9
No	720	94.1
Ratio of drug intake in the past month		
≥90%	742	97.0
<90%	23	3.0
Initial ART regimen		
AZT/3TC/EFV or NVP	428	56.0
D4T/3TC/EFV or NVP	58	7.6
TDF/3TC/EFV or NVP	229	29.9
Second-line regimens	50	6.5
ART regimen at survey		
AZT/3TC/EFV or NVP	383	50.1
D4T/3TC/EFV or NVP	9	1.2
TDF/3TC/EFV or NVP	310	40.5
Second-line regimens	63	8.2
Province or city		
Beijing	76	9.9
Jilin	74	9.6
Hunan	96	12.6
Guangxi	256	31.5
Sichuan	77	10.1
Guizhou	33	4.3
Yunnan	93	12.2
Xinjiang	60	7.8

### Immunological and virological outcomes

Among the patients, the proportion of a CD4 count of 0–199, 200–349, and ≥350 cells/ul before ART were 45.4%, 37.4%, and 17.2%, respectively. After 13.9 months of treatment, the proportion of a CD4 count of ≥350 cells/ul was increased to 37.4%. The median CD4 count before ART increased from 222 (IQR, 215) cells/ul to 303 (IQR, 258) cells/ul at the time of the survey. The great majority of patients (700/765, 91.5%) had a plasma HIV viral load <1000 copies/ml. Among the individuals with VLF, 64 were successful genotyped, and of these, 33 had one or more HIVDR mutations. However, the proportion of viral load failure among patients from Sichuan and Guizhou were 27% and 24%, respectively, which was higher than patients from other provinces.

### HIV drug resistance and subtype

Among the 33 patients identified with HIVDR mutations, all patients harbored HIV-1 strains resistant to non-nucleoside reverse transcriptase inhibitors (NNRTIs) (Table 2). 81.8% of these patients were also resistant to nucleoside reverse transcriptase inhibitors (NRTIs), but only 3% were resistant to both NNRTIs and PIs. The most common NNRTIs mutations were K103N (54.5%), G190A/S/R/ (27.3%), and Y181C (24.2%) in the reverse transcriptase (RT) region. NRTIs mutations were most frequently seen as M184I/V (66.7%) and K65R (39.4%) in the RT region, while PI mutations were only found to be M46L in the protease (PR) region. Among 65 patients with VLF, 56.9% (37/65) patients were subtypes of 01AE, 21.5% (14/65) were 07BC, and the rest were other subtypes. There was no corrections between the drug resistance and the subtype (P>0.05).

**Table 2:** HIV drug resistance mutations among HIV patients with drug resistance.

Antiretroviral drug	N(%)	HIV drug resistance mutations, N (%)
**Total**	33 (100)	
**Non-nucleoside reverse transcriptase inhibitors (NNRTIs, any)**	33 (100)	
**Efavirenz (EFV)**	33 (100)	K103N/KN,18 (54.5)
**Nevirapine (NVP)**	33 (100)	G190A/S/R/GS/RS/AG,9 (27.3)
**Etravirine (ETR)**	21 (63.6)	Y181C/CY,8 (24.2)
**Rilpivirine (RPV)**	24 (72.7)	V179D/T/E,7 (21.2)
		K101E/N/EK,5 (15.2)
		V90I/IV,5 (15.2)
		V106M/IV,5 (15.2)
		V108I/IV,3 (9.1)
		E138A/EG/EK,3 (9.1)
		M230L,3 (9.1)
		L100I,3 (9.1)
		K238N/KT,2 (6.1)
		A98G/AG,2 (6.1)
		Y188FHLY/L,2 (6.1)
		P225HP/H,2 (6.1)
		H221HY,1 (3.0)
		F227FL,1(3.0)
**Nucleoside reverse transcriptase inhibitors (NRTIs, any)**	27 (81.8)	
**Emtricitabine (FTC)**	25 (75.8)	M184I/V/IM,22 (66.7)
**Lamivudine (3TC)**	25 (75.8)	K65R/KR,13 (39.4)
**Abacavir (ABC)**	26 (78.8)	D67N/G/DN,6 (18.2)
**Didanosine (DDI)**	22 (66.7)	Y115F,5 (15.2)
**Stavudine (D4T)**	19 (57.6)	K70E/R,3 (9.1)
**Tenofovir (TDF)**	18 (54.6)	T215FIST/F,3 (9.1)
**Azidothymidine (AZT)**	4 (12.1)	A62V/AV,2 (6.1)
		L74I/IL,2 (6.1)
		V75M/IV,2 (6.1)
		T69N,1 (3.0)
		M41LM,1 (3.0)
		K219E,1 (3.0)
**Protease inhibitors (PIs, any)**	1 (3.0)	
**Tipranavir (TPV)**		M46L,1 (3.0)
**Fosamprenavir (FPV)**		
**Lopinavir (LPV)**		
**Nelfinavir (NFV)**	1 (3.0)	
**Atazanavir (ATV)**		
**Darunavir (DRV)**		
**Indinavir (IDV)**		
**Saquinavir (SQV)**		
**Multi-drug resistance to NNRTI and NRTI**	27 (81.8)	

### Patient characteristics associated with HIV drug resistance

The risk factors for HIVDR that were significant in the univariate logistic analysis were included in the multivariate logistic regression (Table 3). According to the univariate logistic regression model, five potential factors correlated with HIV drug resistance. In the multivariate model, the following two factors were independently correlated with HIVDR: the rate of HIVDR among patients with <90% of drug intake in the past month were 6.0 folds higher than in patients with ≥90% of drug intake (95%CI: 1.7–20.7; P = 0.005), and Sichuan and Guizhou Provinces were 7.3 times higher than the other provinces (95%CI: 3.6–15.2; P<0.0001).

**Table 3:** Factors associated with HIV drug resistance among patients receiving ART in China.

Variable	Number	Drug resistance, N (%)	Crude OR(95%CI)	P-value	Adjusted OR(95%CI)	P-value
Total	765	33 (4.3)				
Age (years)						
<45	416	22 (5.3)				
≥45	349	11 (3.2)	0.6 (0.3–1.2)	0.15		
Sex						
Male	546	26 (4.8)				
Female	219	7 (3.2)	0.7 (0.3–1.5)	0.34		
Ethnicity						
Han	607	27 (4.4)				
Minorities	158	6 (3.8)	0.8 (0.3–2.1)	0.72		
Education						
Junior high school or lower	541	27 (5.0)				
High school or higher	224	6 (2.7)	0.5 (0.2–1.3)	0.16		
Married						
Yes	408	19 (4.7)				
No	357	14 (3.9)	0.8 (0.4–1.7)	0.62		
Registered residence						
City	421	19 (4.5)				
Rural	344	14 (4.1)	0.9 (0.4–1.8)	0.76		
Occupation						
Farmer	270	10 (3.7)				
Other	495	23 (4.6)	1.3 (0.6–2.7)	0.54		
Economics						
Poor	182	11 (6.0)				
General or good	583	22 (3.8)	0.6 (0.3–1.3)	0.19		
Medical insurance						
Yes	665	32 (4.8)				
No	100	1 (1.0)	0.2 (0.03–1.5)	0.11		
Social support						
No	59	8 (13.8)				
Yes	706	25 (3.5)	0.2 (0.1–0.5)	0.001		
Living condition						
Satisfactory	527	25 (4.7)				
Not satisfactory	238	8 (3.4)	0.7 (0.3–1.6)	0.39		
Alcohol in the past 6 months						
No	473	22 (4.7)				
Yes	292	11 (3.8)	0.8 (0.4–1.7)	0.56		
Smoking (cigarettes/day)						
<10	613	25 (4.1)				
≥10	152	8 (5.3)	1.3 (0.6–3.0)	0.52		
Went outside for work in the past three months (days)						
<30	595	26 (4.4)				
≥30	170	7 (4.1)	0.9 (0.4–2.2)	0.89		
Weight						
Increased	224	7 (3.1)				
Decreased	71	6 (8.5)	2.9 (0.9–8.8)	0.07		
No change	470	20 (4.3)	1.4 (0.6–3.3)	0.47		
HIV transmission route						
Heterosexual intercourse	517	25 (4.8)				
Homosexual intercourse	147	3 (2.0)	0.4 (0.1–1.4)	0.15		
Drug injection	65	3 (4.6)	1.0 (0.3–3.2)	0.94		
Other	36	2 (5.6)	1.2 (0.3–5.1)	0.85		
Traditional Chinese Medicine treatment						
Yes	53	3 (5.7)				
No	712	30 (4.2)	0.7 (0.2–2.4)	0.6		
Obtain medicine						
Myself	739	29 (3.9)				
Others	26	4 (15.4)	4.5 (1.4–13.8)	0.01		
Reminded of taking Antiretroviral drug						
Telephone	434	14 (3.2)				
Others	331	19 (5.7)	1.8 (0.9–3.7)	0.09		
Taking medicine in the workplace						
Convenient	274	10 (3.7)				
Inconvenient	136	7 (5.2)	1.4 (0.5–3.9)	0.48		
Unemployed	355	16 (4.5)	1.2 (0.6–2.8)	0.59		
Side reaction in the past month						
Small side reaction	743	31 (4.2)				
Big side reaction	22	2 (9.1)	2.3 (0.5–10.3)	0.28		
Ratio of drug intake in the past month (%)						
≥90	744	29 (3.9)				
<90	21	4 (19.0)	5.8 (1.8–18.3)	0.003	6.0(1.7–20.7)	0.005
Ratio of on-time drug intake in the past month						
≥90%	748	29 (3.9)				
<90%	17	4 (23.5)	7.6 (2.3–24.8)	0.001		
Missed doses in the past month						
Yes	45	1 (2.2)				
No	720	32 (4.4)	2.0 (0.3–15.3)	0.49		
Initial ART regimen						
Regimens without TDF	497	21 (4.2)				
Regimens with TDF	268	12 (4.5)	1.1 (0.5–2.2)	0.87		
ART regimen at survey						
Regimens without TDF	410	14 (3.4)				
Regimens with TDF	355	19 (5.4)	1.6 (0.8–3.2)	0.19		
Place of residence						
Others	655	16 (2.4)				
Sichuan and Guizhou	110	17 (15.5)	7.3 (3.6–14.9)	<0.001	7.4 (3.6–15.2)	<0.0001

## Discussion

In this study, we analyzed HIVDR data in order to evaluate the prevalence and risk factors among 765 patients who were undergoing treatment. Our findings showed that 91.5% (700/765) achieved virological suppression (VL<1000 copies/ml) after 13.9 months of ART, which is better than the outcomes of previous surveillance studies in China [19–21]. 33 patients (4.3%) had verified HIVDR which is slightly lower than other countries where national free ART is available. The prevalence of HIVDR in Cameroon and Namibia were 5.3% and 5% respectively [22, 23] but significantly higher than Malawi [24] of 3.4%. Our results suggest that China’s free ART program is providing high quality care to HIV/AIDS patients. There are several reasons to explain the low virological failure and drug resistance among HIV-infected patients receiving first-line ART. First, medical care is accessible at many levels of the health systems including provincial, prefecture, and county hospitals. Most care is provided at the community level and through outreach, with telephone calls or home visitation. Second, all doctors and health staff involved in providing ART and care management receive additional training [25].

Although our study showed that China has met the WHO target for 90% of patients having their viral load suppressed, as HIV/AIDS patients live longer and are on ART for life, the number of patients with drug resistance is likely to increase. A combination of strategies is required to combat drug resistance. New medicines that can more robustly cope with drug resistance mutants are needed, especially for those with common mutations such as K103N and M184I/ V [19, 26, 27]. M184I/V confers resistance to lamivudine, which is often the first mutation to develop in patients receiving partially suppressive triple combination therapy including lamivudine [28]. K103N is one of the most frequent mutations conferring resistance to most available NNRTIs [29].

Factors independently associated with the incidence of HIVDR were: the ratio of drug intake in the past month; and place of residence. The first factor reflected that adherence was a direct factor causing HIVDR, with 21(2.7%) patients having reported to have lower than 90% of drug intake in the past month. Similar findings had been reported in our previous studies in China [19, 27, 30], where poor adherence clearly leads to the occurrence of HIVDR. Good adherence can suppress plasma HIV RNA and utilize the optimum effectiveness of the ART therapy. Several studies have focused on strategies to improve adherence, including social support [31, 32], behavioral interventions [33], contingency management strategies [34], directly administered antiretroviral therapy (DAART) [35], and technological interventions [36]. The Chinese strategy focuses on education and counseling to improve the adherence of patients; but a comprehensive strategy using some of the other interventions is needed.

The reasons why patients from Sichuan and Guizhou had worse outcomes compared to patients from other provinces was unclear. We found, however, that the composition of patients from this population differed compared to patients from other provinces. Their differential risk for drug resistance may have been mediated through factors influencing adherence such as education, economic level, healthcare providers support, and adherence to ART. 78.2% of the Sichuan and Guizhou patients had received middle school education or lower compared with 69.5% in other provinces, 32.7% of patients in Sichuan and Guizhou were poor versus 22.3% s in other provinces, 59.1% of patients in Sichuan and Guizhou get support from healthcare providers compared with 89.9% in other provinces, and 14.5% in Sichuan and Guizhou had a poor adherence to ART compared with 7.8% in other provinces. There may, however, have been unmeasured confounding variables that led to this observation. Observations about drug resistance found on the population level, may not apply to individual patients. Future studies should explore health systems and individual level differences to better elucidate why resistance was greater in Sichuan and Guizhou provinces than other provinces.

We also found that regimens with or without TDF showed the same results in causing HIVDR, which needs further investigation. TDF is preferred to its predecessors AZT and d4T in the ART program because of its better safety profile [37] which has been recommended by WHO for HIV first-line treatment. Researchers found that patients on TDF-based first-line regimens had fewer drug-resistant mutations [38]. With the scale-up of TDF, evidence on this issue tends to show different results concerning mortality, the CD4 cell count, and virological failure [38–40]. In our study, patients at the survey on regimens with and without TDF were 5.4% (19/355) and 3.4% (14/410), respectively (P = 0.19). Further studies are needed to clarify the effects of regimens with TDF in China.

This study has some limitations. First, this was a cross-sectional study and patients who terminated treatment (due to adverse reactions, loss to follow-up, or death) would not have been sampled, which may have led to overestimated treatment effectiveness. Second, adherence was assessed by self-reporting of having missed doses in the past month, which may not reflect the true adherence. Finally, we found the drug resistance difference between Guizhou, Sichuan, and other provinces. Explaining the differences focused on the socioeconomic status of the inhabitants, but in fact transmitted drug resistance and possible presence of transmission clusters with HIV drug resistant variants may differ in different provinces which we do not know.

In conclusion, a representative national sample of HIVDR surveillance across China demonstrated excellent virological and immunological outcomes at 9–18 months among patients receiving first-line ART treatment. However, poor adherence to treatment is still a key problem regardless of the efforts on the regimens, which has led to the high rate of HIVDR. Drug resistance widely varies among provinces. More research needs to focus on the adherence of patients and long-term studies monitoring drug resistance should be completed in some select cases.

## Supporting Information

S1 File Questionnaire of this research. (DOC)

S1 Table Relevant data underlying the findings described in manuscript. (SAS7BDAT)
