# Supplementary material for: Drug Resistance and Virological Failure among HIV-Infected Patients after a Decade of Antiretroviral Treatment Expansion in Eight Provinces of China
Source: PLoS One. 2016 Dec 20;11(12):e0166661. doi: 10.1371/journal.pone.0166661 (PMC5172524; doi:10.1371/journal.pone.0166661)
Supplement: S1 File — (DOC) [file pone.0166661.s001.doc]

**Questionnaire of Chinese language**

1. 调查时间： 年 月 日
2. 姓名（同抗病毒治疗库一致）： 身份证号码：

□□□□□□-□□□□□□□□-□□□□

1. 抗病毒治疗前，最后一次CD4计数： ；检测时间： 年 月 日
2. 开始抗病毒治疗时间（同国家HIV抗病毒治疗库一致）： 年 月 日
3. 开始抗病毒治疗方案： + +
4. 目前抗病毒治疗方案： + + 停服抗病毒药物□
5. 性别：①男□ ②女□
6. 民族：①汉族□ ②其他□（ 族）
7. 文化程度: ①文盲□ ②小学□ ③初中□ ④高中□ ⑤大专或本科□ ⑥研究生及以上□
8. 户口：①农村户口□ ②城镇户口□
9. 职业情况：①务农为主□ ②外出打工为主□ ③经商或个体户□ ④企业或公司上班□ ⑤机关或事业单位□ ⑥其他□，说明：
10. 您的婚姻及性伴情况？ ①在婚□ ②同居□ ③未婚□ ④其他□
11. 您感染HIV的途径：①异性□ ②同性□ ③吸毒□ ④其他□
12. 您的配偶或固定性伴是否感染HIV：①是□ ②否□ ③无配偶或固定性伴□
13. 您的配偶或固定性伴是否接受过艾滋病抗病毒治疗（西药）：①是□ ②否□ ③无配偶或固定性伴□
14. 您是否接受过**抗艾滋病病毒的中药治疗**（不包括一般的中药治疗）：①是□ ②否□
15. 您认为您的家庭经济状况： ①贫困□ ②一般□ ③小康□ ④富裕□
16. 您现在是否有医疗保险（包括公费、农村合作医疗等）？ ①是□ ②否□
17. 在需要时，您获得的社会与情感支持的频率如何（包括各种来源的支持）？
    ①总是□ ②经常□ ③有时□ ④从来没有□
18. 总的来说，您对您的生活是否满意？
    ①非常满意□ ②满意□ ③不满意□ ④非常不满意□
19. 近半年内您饮酒（包括含酒精的饮料）的频率？ ①从不喝酒□ ②每月1次或以下□ ③每月2-3次□ ④每周1-3次□ ⑤每周4-6次□ ⑥每天□
20. 近半年内您平均每天吸多少支烟？ 支
21. 在过去3个月内，您外出打工多少天？ 天
22. 近一年来，您的体重是否有变化：①增加□ ②减少□ ③无变化□

**读给调查对象听：**许多人服用抗病毒药物会面临一些困难， 如忘记、农忙或工作忙什么原因等，下面就问一些服药情况。

1. 您最近一次由谁领抗病毒药物：①本人□ ②他人代领□
2. 您最近一次从哪儿领抗病毒药物**（单选）**: ①村卫生室□ ②乡镇卫生院 □ ③县或市疾病控制中心□ ④县或市医院□ ⑤其他 □，请说明：
3. 您最近两次领取药物的间隔时间是： 天
4. 近半年内，您去领一次抗病毒药物需多少分钟（往返路程和领药总时间）？
    分钟
5. 近半年内，在您领药时，医护人员（包括发药者、医生）是否会询问您服药情况、提供服药帮助？
   ①每次□ ②大多时候□ ③有时会，有时不会□ ③几乎没有□
6. 您如何提醒服用抗病毒药物（**单选**）： ①手机□ ②闹钟□ ③家人/亲友□ ④同伴督导员（其他患者）□ ⑤其他□： **⑥无□**
7. 您在工作单位服用抗病毒药物？ ①方便□ ②不方便□ ③无工作□
8. 您在住所服用抗病毒药物？ ①方便□ ②不方便□ ③无住所□
9. 在**近**1个月内，您服用抗病毒药物的副反应情况如何？ ①没有或几乎没有□ ②副反应小□ ③副反应比较大□ ④副反应很大□
10. 目前服用抗病毒药物情况： ①维持初始一线方案□ ②更换其他一线药物□ ③更换二线方案□ ④停服药物□
11. 在最近的1个月内，您服用全部抗病毒药物的比例（0％表示没有服用、100％表示全部服用）：

| 0% | 10% | 20% | 30% | 40% | 50% | 60% | 70% | 80% | 90% | 95% | 100% |
| --- | --- | --- | --- | --- | --- | --- | --- | --- | --- | --- | --- |
|  |  |  |  |  |  |  |  |  |  |  |  |

1. 在最近的1个月内，您按时服用抗病毒药物的比例（0％表示完全不按时、100％表示全部按时）：

| 0% | 10% | 20% | 30% | 40% | 50% | 60% | 70% | 80% | 90% | 95% | 100% |
| --- | --- | --- | --- | --- | --- | --- | --- | --- | --- | --- | --- |
|  |  |  |  |  |  |  |  |  |  |  |  |

1. 在近1个月内，您有没有错服（包括多服、漏服）抗病毒药物？ ①是□ ②否□
2. 您最近1次漏服抗病毒药物在什么时间？ ①一周以内□ ②1－2周前□ ③2－4周前□ ④1－3个月前□ ⑤3个月前□ ⑥从来没有漏服□

漏服主要在星期几？

**访谈员：**①医院医生□ ②医院护士□ ③疾控中心工作人员□ ④其他□，请说明：

**填写人签名： 日期： ；审核人签名： 日期：**

**Questionnaire of English language**

1. Therapeutic agency code：□□□□□□-□□□
2. Investigation of time：year  month  day
3. Name: ID card:
4. The last CD4 count before ART: Date: year  month  day
5. The time of starting ART: year  month  day
6. Initial HAART regimens: + +
7. HAART regimens at survey: + +
8. Sex: ①male□ ②female□
9. Ethnicity: ①han□ ②minorities□（ minority）

10.Education: ①illiterate□ ②primary□ ③junior middle school□ ④high school□ ⑤undergraduate□ ⑥Graduate student or more□

1. Registered residence: ①rural□ ②city□
2. Occupation: ①farmer□ ②worker□ ③bussinessman□ ④Enterprise or company to work□ ⑤institution□ ⑥other□，detail：
3. Your marriage and sexual partners? ①In marriage□ ②cohabitation□ ③unmarried□ ④other□
4. Route of infecting HIV：①heterosexual□ ②Homosexual□ ③drug injection□ ④other□
5. Do you have a spouse or a partner with HIV: ①yes□ ②no□ ③No spouse or partner□
6. Does your spouse or partner receive HIV / AIDS antiviral therapy (Western Medicine)：①yes□ ②no□ ③No spouse or partner□
7. Have you accepted the anti HIV Chinese medicine treatment (does not include the general traditional Chinese medicine treatment)：
8. yes□ ②no□
9. what is your family's economic situation：
10. poor□ ②commonly□ ③well off□ ④affluent□

19. Do you have health insurance now (including rural cooperative medical fees, etc.)？ ①yes□ ②no□

20. When needed, how do you get the frequency of social and emotional support (including support from a variety of sources)？
①always□ ②often□ ③sometimes□ ④never□

21. Are you satisfied with your life?

①Very satisfied□ ②satisfied□ ③Dissatisfied□ ④very Dissatisfied□

22. What is the frequency of drinking alcohol (including alcoholic drinks) in the past six months? ①never□ ②one time every month□ ③2-3 times every month□ ④1-3 times every week□ ⑤4-6times every week□ ⑥everyday□

23. How many cigarettes do you smoke every day in the past six months?

24. How many days do you go out for work in the past three months?

Days.

25. Do you have any change in your weight in the past year?

① increase□ ②reduce□ ③no change□

26 Who received your latest antiretroviral drugs?

①himself/herself□ ②Other people□

27. Where did you get the antiretroviral drugs from the last time?

①village clinic□ ②Town hospital □ ③County CDC□ ④County hospital□ ⑤other □，statement：

28.What is the time interval between the last two times of getting ART? Days

29. How many minutes would you take to get the ART drugs (back and forth) in the past six months. Minutes

30.Did healthcare providers ask you about the situations of taking medicine and providing help?

①every-time□ ②always□ ③sometimes□ ③never□

31.how to remind taking ART drugs?

①telephone□ ②clock□ ③family□ ④other patients□

⑤other□： **⑥no□**

**32.IS it convenient to take drugs at your workplace?**

①convenience□ ②inconvenience□ ③no job□

33.Is it convenient to take drugs at home?

①convenience□ ②inconvenience□ ③no job□

34. What’s the adverse effect of taking ART drugs in the past month?

①no□ ②little side effects□ ③Relatively large side effects□ ④Great side effects□

35.HAART regimens at survey: ①first-line regimens□ ②change into other first-line regimens□ ③change into second-line regimens□ ④stop medicine□

36. Ratio of drug intake in the past month? （0％ means stop、100％means taking all the drugs）

| 0% | 10% | 20% | 30% | 40% | 50% | 60% | 70% | 80% | 90% | 95% | 100% |
| --- | --- | --- | --- | --- | --- | --- | --- | --- | --- | --- | --- |
|  |  |  |  |  |  |  |  |  |  |  |  |

37. Ratio of on-time drug intake in the past month? （0％ means never take drug on time、100％means taking all the drugs on-time）

| 0% | 10% | 20% | 30% | 40% | 50% | 60% | 70% | 80% | 90% | 95% | 100% |
| --- | --- | --- | --- | --- | --- | --- | --- | --- | --- | --- | --- |
|  |  |  |  |  |  |  |  |  |  |  |  |

38. have you missed the doses in the past month? ①yes□ ②no□

39. what’s the last time of missing doses? ①within one week□ ②before 1-2 weeks□ ③before 2－4 weeks□ ④before 1－3months□ ⑤before 3 months□ ⑥never miss□
